# Synergistic Roles of Fimbriae and Gingipain Genotypes in *Porphyromonas gingivalis* and Their Association With Periodontitis Severity

**DOI:** 10.1155/ijod/6236248

**Published:** 2026-04-29

**Authors:** Manohar Kugaji, Kishore Bhat, Uday Muddapur, Ram Surath Kumar, Suman Kumar Ray, Eswar Kandaswamy, Vinayak Joshi

**Affiliations:** ^1^ Central Research Laboratory, Maratha Mandal’s Nathajirao G. Halgekar Institute of Dental Sciences and Research Centre, Belagavi, 590010, Karnataka, India; ^2^ Arihant Hospital, Belagavi, 590010, Karnataka, India; ^3^ Department of Biotechnology, KLE’s BVB College of Engineering and Technology, Hubballi, 580031, Karnataka, India; ^4^ Department of Public Health Dentistry, KLE Vishwanath Katti Institute of Dental Sciences, KLE Academy of Higher Education and Research, Belagavi, 590010, India; ^5^ Department of Periodontics, School of Dentistry, Louisiana State University Health Sciences Center, New Orleans, Louisiana, USA, lsu.edu

**Keywords:** clinical severity, fimbriae, gingipain genotypes, *p. gingivalis*, periodontitis, virulence

## Abstract

**Objective:**

*Porphyromonas gingivalis* is a major periodontal pathogen periodontitis, with virulence mediated by fimbriae and gingipains. Differences in virulence may influence disease severity. This study aimed to assess the association and co‐occurrence of fimbriae and gingipain genotypes and their relationship with clinical severity in periodontitis.

**Materials and Methods:**

This secondary analysis included 120 subgingival plaque samples from patients with periodontitis. Fimbriae (*fimA* types I–V) and gingipain (*kgp*, *rgpA*) genotypes were identified using PCR and restriction enzyme digestion, and *P. gingivalis* load was quantified by real‐time PCR. Associations between genotypes and clinical parameters (probing depth and clinical attachment loss) were evaluated using Spearman’s correlation and chi‐square tests. Binary logistic regression assessed the association between periodontal disease severity and the presence of a combined virulence genotype, reported as odds ratios (ORs).

**Results:**

The *fimA* types II and III and gingipain genotypes *kgp-I* and *kgp-II* were significantly associated with deeper PD and greater CAL (*p*  < 0.05). *fimA* type II was the most prevalent across all bacterial load percentiles, followed by type IV. *kgp-I* and *rgpA* type A were correlated with higher *P. gingivalis* counts. Significant positive correlations were observed between fimbriae and gingipain genotypes (*p*  < 0.05). Patients with CAL ≥5 mm had significantly higher odds of harboring the combined virulence genotype than those with CAL <5 mm (OR = 3.56; 95% CI: 1.43–8.47; *p* = 0.011).

**Conclusion:**

Specific fimbriae and gingipain genotypes co‐occur and are linked to increased bacterial load suggesting synergistic roles in the pathogenicity of *P. gingivalis*. The findings support the hypothesis that these virulence factors act synergistically to influence disease severity.

**Clinical Relevance:**

The integration of microbial virulence profiling with host immune response characterization may improve risk stratification and enable the development of personalized periodontal care strategies. Furthermore, microbial genotypic profiling may support the identification of disease‐specific targets, thereby facilitating the implementation of tailored therapeutic interventions for effective periodontitis management.

## 1. Introduction

Periodontitis is an inflammatory condition affecting the tissues that support the teeth, marked by the loss of clinical attachment and alveolar bone, which can result in tooth loss [1, 2]. *Porphyromonas gingivalis* (*P. gingivalis*) is a key pathogen associated with periodontal disease, implicated in the initiation and progression of periodontitis [3, 4]. *P. gingivalis* has fimbriae on its surface that assist the pathogen in adhering to the receptors of gingival epithelial cells, facilitating initial attachment and biofilm formation [4, 5]. Gingipains, a class of cysteine proteinases, account for most (~85%) of its proteolytic activity [6, 7]. Fimbriae are known to activate cells, leading to the release of cytokines that trigger an inflammatory response at the infection site [8]. Beyond epithelial adherence, fimbriae contribute to the coaggregation with various plaque‐forming bacteria such as *Actinomyces naeslundii*, *Streptococcus gordonii*, and *Streptococcus oralis*, thereby aiding biofilm formation and promoting dysbiosis [2].

Based on the nucleotide variation, fimbriae are classified into six genotypes (type I, Ib, II, III, IV, and V) and are encoded by the *fimA* gene [9]. Gingipains are trypsin‐like cysteine proteinases and are broadly categorized as arginine‐dependent gingipain R (protein Rgp) and lysine‐dependent gingipain K (protein Kgp) based on their ability to digest polypeptides after arginine and lysine residues, respectively [10]. The gene coding for gingipain R containing hemagglutinin/adhesion (HA) domain is called *rgpA*, whereas the gene that encodes gingipain R without an HA domain is referred to as *rgpB*. Allaker et al. [11] recognized the three subtypes of *rgpA* gingipain (type A, B, and C) based on the nucleotide variation. The gene encoding gingipain K is referred to as *kgp*. Beikler et al. [12] reported two diverse types of *kgp* (*kgp-I* and *kgp-II*) based on the sequence dissimilarity in the region that encodes the catalytic domain. Gingipains can selectively degrade adhesion molecules on gingival epithelial cells, increasing epithelial permeability and allowing bacterial products such as lipopolysaccharide and proteoglycan to penetrate the tissue, thereby triggering an inflammatory response [7, 13]. Additionally, gingipains alter capillary permeability, impede blood coagulation, and increase bleeding in periodontal tissues [6, 7].

Previous research has demonstrated that both fimbriae and gingipain contribute to the coaggregation and interactions among diverse microbial species within the oral cavity [14, 15]. Moreover, the presence of the gingipain genotype has been linked to the expression *fimA* gene, which is critical for fimbriae maturation [14, 16]. These findings suggest that complex interactions between these two virulence factors may underlie their capacity to initiate biofilm formation, thereby facilitating disease progression. Variations in strain‐specific virulence among *P. gingivalis* may result in differences in disease severity. Therefore, evaluating patients based on the presence of distinct virulence genotypes could provide critical insights into these interactions and their role in disease progression. Extensive research on fimbriae and gingipain genotypes highlights their importance in the development and progression of periodontitis [17–21], making them key targets for further studies and potential therapeutic interventions. However, the relationship between fimbriae and gingipain and their potential impact on clinical disease severity remains poorly investigated. Understanding this association may help researchers and clinicians elucidate disease etiology and develop new treatment strategies for periodontitis management.

The study evaluated the association between fimbriae and gingipain genotypes and the levels of *P. gingivalis* in subgingival plaque samples and determined their relationship with periodontitis. The study also assessed whether combinations of these genotypes were associated with clinical periodontal parameters, including probing depth and clinical attachment loss. The hypothesis was that specific fimbriae and gingipain genotypes, individually and in combination, are associated with higher levels of *P. gingivalis* and greater clinical severity of periodontitis.

## 2. Materials and Methods

### 2.1. Study and Participants

This secondary analysis utilized data from a previously published clinico‐microbiological study on periodontitis, which included 120 subgingival plaque samples processed for fimbriae and gingipain genotyping [22–24]. All subjects provided written consent prior to the sample collection. Detailed protocols for participant recruitment, sample collection, DNA extraction, quantitative real‐time polymerase chain reaction (PCR), and genotyping have been described elsewhere [22–24]. Briefly, participants were enrolled according to the 1999 American Academy of Periodontology guidelines [25]. Inclusion criteria were age of at least 18 years, a minimum of 20 teeth, bleeding on probing, probing depth (PD) of 5 millimeters or greater, and clinical attachment loss (CAL) of 1 millimeter or greater at four or more sites. Individuals with systemic disease, pregnancy, smoking habits or consumed smokeless tobacco, on recent antibiotic or dental therapy within 3 months were excluded. Ethical approval for this study was obtained from the Institutional Review Board (Certificate No. 2016/819).

### 2.2. Sample Processing and Analysis

Collection and processing of subgingival plaque samples, DNA extraction, and quantitative real‐time PCR were described previously [22]. Primers for *fimA* genotypes (types I–V) and gingipain genes (*kgp*, *rgpA*) were identical to those reported earlier [23, 24]. Restriction digestion was performed to detect the *kgp* genotypes (*kgp-I* and *kgp-II*) by using restriction enzyme Fastdigest *MseI* (Thermo Scientific, Waltham, MA, USA) [12] and *rgpA* genotypes (types A, B, and C) by using Fastdigest *RsaI* (Thermo Scientific, Waltham, MA, USA) [11]. For this secondary analysis, genotype data and clinical parameters were analyzed to explore associations between microbial virulence factors and periodontal disease characteristics.

### 2.3. Statistical Analysis

Statistical analysis was conducted using SPSS software version 26 (IBM Corp). The association between different levels of periodontal disease severity and the presence of fimbriae and gingipain genotypes was examined with the chi‐square test. Additionally, the relationship between the presence of different genotypes of fimbriae and gingipain was assessed using Spearman’s rank correlation coefficient. Binary logistic regression analysis was used to evaluate the relationship between periodontal disease severity, categorized by clinical attachment loss (CAL ≥5 mm and <5 mm), and the presence of a combined virulence genotype. The strength of association was quantified using odds ratios (ORs). A *p*‐value of ≤ 0.05 was considered statistically significant for all tests.

## 3. Result

Variable"?>A total of 120 individuals with periodontitis were included (59 males and 61 females; mean age, 45 years) [22–24]. All the patients included in the study had generalized moderate to severe periodontitis with CAL ≥ 3 mm. The mean PD was 5.68 mm, and the mean CAL was 5.20 mm. The distribution and association among fimbriae and gingipain genotypes are presented in Table 1. Significant positive correlations were observed between the following genotype pairs; *fimA* type I and *rgpA* type B; *fimA* type II and *kgp-I*; *fimA* type II and *rgpA* type A; *fimA* type IV and *kgp-II*; *fimA* type IV and *rgpA* type A (Spearman’s correlation, *p*  < 0.05).

**Table 1:** Correlation between fimbriae and gingipain genotypes in patient with periodontitis patients.

Variable		*kgp*		*rgpA*		
		I	II	A	B	C
*fimA*	I	0.04	0.14	−0.03	0.333 ^∗∗^	−0.08
	Ib	−0.02	0.13	0.10	0.02	−0.04
	II	0.358 ^∗∗^	0.03	0.298 ^∗∗^	0.13	0.02
	III	0.12	−0.08	−0.03	0.14	−0.02
	IV	0.06	0.200 ^∗^	0.276 ^∗∗^	−0.11	0.11
	V	NA	NA	NA	NA	NA

*Note:* Spearman’s rank correlation coefficients between fimbriae and gingipain genotypes.

^∗^ *p*  < 0.05 indicates statistical significance.

^∗∗^ *p*  < 0.01 indicates high significance.

The distribution of combined virulence (Cv) genotypes with the combination of *fimA*:*kgp:rgpA* was studied and found that total of 10 Cv genotypes in the periodontitis cases (Table 2). The genotypes IV:I:A (30.5%) and II:II:A (25.2%) were detected more frequently, whereas the combination of II:I:C and II:II:C (1% each) was less frequently found in the periodontitis.

**Table 2:** Distribution of combined virulence (Cv) genotypes of *P. gingivalis* in chronic periodontitis patients.

Combined genotypes *fimA*:*kgp:rgpA*	*n* (%)
I:I:A	17 (17.9)
Ib:I:A	6 (6.3)
II:I:A	13 (13.6)
II:I:B	12 (12.6)
II:II:A	24 (25.2)
II:II:B	4 (4.2)
II:I:C	1 (1.0)
II:II:C	1 (1.0)
III:II:C	2 (2.1)
IV:I:A	29 (30.5)

*Note:* All values are expressed as a frequency with percentages (in parentheses)

The association between combined genotypes and clinical parameters was analyzed by Pearson’s chi‐square test (Table 3). Combined virulence of fimbriae and gingipain was significantly associated with different categories of PD (*p* = 0.040) and CAL (*p*‐ = 0.005), with the highest presence seen in a PD range of 5–6 mm and CAL ≥ 5 mm.

**Table 3:** Association of combined virulence (Cv) genotypes with clinical parameters in periodontitis.

Variable	Combined virulence (Cv) genotypes			*χ* ^2^	*p*‐Value
	Absent *n* (%)	Present *n* (%)	Total *n* (%)		
Periodontal pocket					
3–5 mm	0 (0.0%)	7 (100%)	7 (100%)	8.310	0.040 ^∗^
5–6 mm	25 (26.3%)	70 (73.7%)	95 (100%)		
6–7 mm	0 (0.0%)	14 (100%)	14 (100%)		
>7 mm	0 (0.0%)	4 (100.0%)	4 (100%)		
Total	25 (20.8%)	95 (79.2%)	120 (100%)		
Clinical attachment loss (CAL)					
<5 mm	14 (35.9%)	25 (64.1%)	39 (100%)	7.950	0.005 ^∗^
≥5 mm	11 (13.6%)	70 (86.4%)	81 (100%)		
Total	25 (20.8%)	95 (79.2%)	120 (100%)		

*Note:* Pearson’s chi‐square test was used to assess associations between combined genotypes and clinical parameters (probing depth and clinical attachment loss).

^∗^ *p* ≤ 0.05 indicates statistical significance.

**Figure 1:**
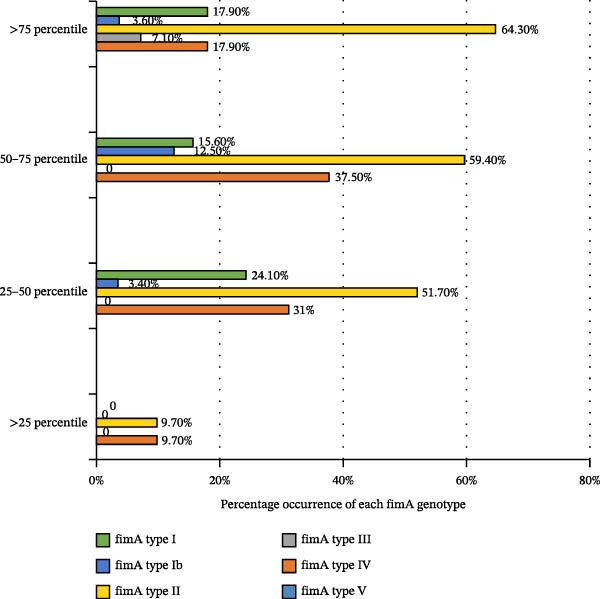
Percentage distribution of *fimA* genotypes (types I–V) across four *P. gingivalis* load categories: >75th percentile (>20.40 × 10^3^ DNA copies), 50–75th percentile (3.30–20.40 × 10^3^), 25–50th percentile (0.04–3.30 × 10^3^), and <25th percentile (<0.04 × 10^3^).

**Figure 2:**
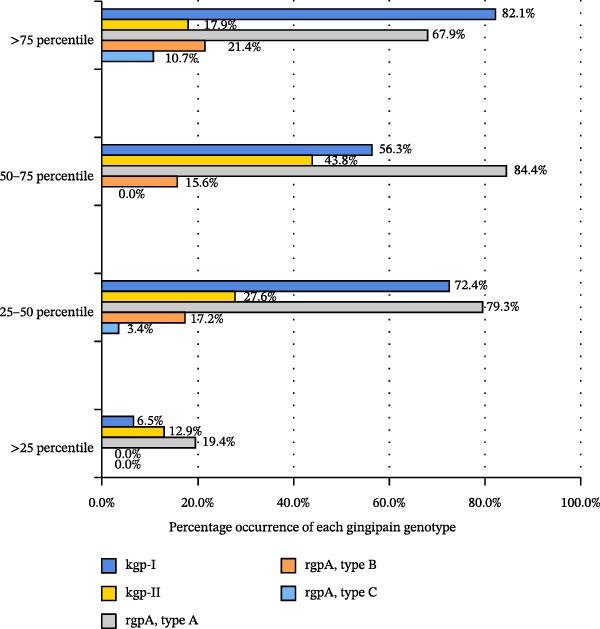
Percentage distribution of gingipain genotypes (*kgp-I*, *kgp-II* and *rgpA* types A–C) across four *P. gingivalis* load categories: >75th percentile (>20.40 × 10^3^ DNA copies), 50–75th percentile (3.30–20.40 × 10^3^), 25–50th percentile (0.04–3.30 × 10^3^), and <25th percentile (<0.04 × 10^3^).

The association between the prevalence of fimbriae and gingipain genotypes with the count of *P. gingivalis* is illustrated in Figures 1 and 2. The *P. gingivalis* counts were stratified into four groups based on percentiles: >75th percentile group (>20.40 × 10^3^ DNA copies), 50–75th percentile group (3.30–20.40 × 10^3^ DNA copies), 25–50th percentile group (0.04–3.30 × 10^3^ DNA copies) group and <25th percentile group (<0.04 × 10^3^ DNA copies). *fimA* type II was the most prevalent genotype across all categories, with the highest frequency (64.3%) in the >75th percentile group. The genotype *fimA* type IV followed, with notable prevalence in the 50–75th percentile (37.5%) and 25–50th percentile (31%). Types I and Ib were less frequent, while types III and V were rare (Figure 1).

Among gingipain genotypes, *kgp-I* exhibited the highest prevalence in the >75th percentile group (82.1%), compared to *kgp-II* (17.9%). Similarly, *rgpA* type A showed the highest *P. gingivalis* counts (67.9%, 84.4%, 79.3%, and 19.4% across percentile groups), followed by types B and C (Figure 2).

Binary logistic regression revealed a statistically significant association between disease severity and virulence profile. Patients with CAL ≥5 mm were 3.56 times more likely to present with the combined virulence genotype than patients with CAL <5 mm (OR = 3.56; 95% CI: 1.43–8.47; *p* = 0.011).

## 4. Discussion

The findings confirm significant associations between fimbriae and gingipain genotypes and clinical severity in periodontitis. Previous work has reported the distribution of *fimA* genotypes among individuals with periodontitis and healthy subjects, including studies conducted in Indian populations as well as in Japan, Brazil, Europe, and USA [16–18, 26, 27]. Across these populations, *fimA* types II and IV consistently show higher prevalence in periodontitis cases, whereas type I is more frequently associated with periodontal health. These findings are aligned with our earlier work in an Indian cohort, in which *fimA* types II and IV were significantly associated with chronic periodontitis and type II, in particular, correlated with increased bacterial colonization and deeper periodontal pockets [23].

The findings of the present investigation extend these observations that the combination of fimbriae and gingipain genotypes were significantly associated with greater probing depth and clinical attachment loss, indicating their role in the clinical severity of periodontitis. Furthermore, *fimA* type II emerged as the predominant genotype across bacterial load percentiles. These results are aligned with previous studies identifying *fimA* type II as a highly virulent fimbrial genotype associated with periodontitis [9, 26, 28]. Its presence across all bacterial load percentiles reinforces its significance as an important factor in bacterial colonization and biofilm development. Although *fimA* type IV was detected at a lower frequency compared to type II, its presence aligns with previous report describing a moderate association with disease severity [29].

Analysis of gingipain genotypes indicated that *kgp-I* was more frequently observed in samples with higher bacterial load percentiles, and *rgpA* type A showed notable prevalence in these groups, suggesting a possible link with cases exhibiting greater clinical severity. Importantly, significant positive correlations were observed between *fimA* type II and gingipain genotypes (*kgp-I* and *rgpA* type A), which suggests the coordinated role of fimbriae and gingipain as evidenced by other research studies [14–16]. This interaction is biologically plausible, as gingipains facilitate epithelial adhesion, immune dysregulation, and proinflammatory cytokine release [30]. Mechanistic studies have demonstrated roles for gingipains in cytokine induction, epithelial invasion, and apoptosis [31], contributing to both the degradation of host tissues and the modulation of immune responses [32–34]. Specifically, *kgp-I* has been associated with the invasion of deep tissues, whereas rgpA variants are recognized for their significant involvement in the regulation of inflammatory processes [6, 16]. These observations support the concept that combined virulence determinants may exert compounded effects on periodontal tissue degradation. The genotype *kgp-II* was more frequently detected in the shallow pocket depths and was associated with comparatively less clinical attachment loss. Whereas Beikler et al. [12] did not observe any association between the gingipains and clinical characteristics.

The unique virulent property of *P. gingivalis* is attributed to the combination (Cv types) of specific virulent genotype [27]. The Cv genotypes in the combination of *fimA*:*kgp:rgpA* was analyzed. Interestingly, the most virulent *fimA* genotypes (types II and IV) were more often detected in combination with *rgpA* type A, whereas these same *fimA* types were least frequently associated with *rgpA* type C. The *kgp* genotypes I and II were distributed inconsistently across all genotype combinations, with no clear pattern of association. Similar findings were reported by Yoshino et al. [27], who also observed a preferential association between specific *fimA* and *rgpA* genotypes suggesting a possible linkage or cooperative role in pathogenicity.

The results also align with previous evidence that virulence genotypes of *P. gingivalis* tend to co‐occur rather than distribute randomly. Kristoffersen et al. [14] demonstrated significant associations between the *fimA* and gingipain *rgpB* genotypes, reinforcing the view that integrated virulence networks, rather than isolated genetic markers, may better represent pathogenic potential. Studies further support that gingipain‐mediated tissue invasion and immune impairment are facilitated by fimbriae‐enabled adhesion and co‐aggregation in the biofilm [3, 13]. This co‐occurrence of virulence factors in this study highlights the necessity of evaluating virulence as a coordinated system in determining the pathogenicity of *P. gingivalis*.

From a translational perspective, identifying specific fimbriae and gingipain genotypes may offer clinically relevant value. Genotype profiling of *P. gingivalis* in clinical samples may facilitate risk stratification among patients with periodontitis, allowing early identification of individuals more susceptible to rapid disease progression. Fimbrial and gingipain profiles may also serve as biomarkers for early diagnosis of high‐risk cases. Gingipains have been widely explored as therapeutic targets, with inhibitors designed to block their activity to reduce tissue destruction and immune dysregulation. In addition to their therapeutic potential, recent evidence supports the diagnostic utility of gingipain‐based antigen detection [35–38].

The present study identified significant positive correlations between *fimA* type II and gingipain genotypes *kgp-I* and *rgpA* type A, as well as between *fimA* type IV and both *kgp-II* and *rgpA* type A, highlighting coordinated occurrence of these virulence factors in periodontitis. This finding corroborates previous research [14, 16], which highlights the essential role of gingipain proteolytic activity in facilitating effective fimbrial function [38]. Furthermore, the result suggests that the synergistic interplay between fimbriae and gingipains may potentiate the overall virulence of *P. gingivalis*, thereby impacting its ability to colonize, invade, and contribute to pathogenesis within the oral environment. Further investigation is necessary to understand the factors influencing this relationship.

This study has certain limitations that should be taken into account when interpreting the findings. The sample size, although sufficient to detect significant associations, may limit the generalizability of genotype distribution patterns, particularly given known geographic and ethnic variability in *P. gingivalis* virulence characteristics. Larger, well‐powered studies are needed to confirm the genotypic associations and to clarify the role of each genotype within different virulence gene profiles. However, this study provides valuable preliminary insights and the limited number of cases within specific genotype combinations should be interpreted with caution. The cross‐sectional study design restricts causal inference, preventing the determination of whether specific genotype combinations directly contribute to disease progression over time. In addition, PCR‐based detection identifies the presence of virulent genes but does not account for gene expression levels, protein activity, or post‐translational modifications that influence functional pathogenicity. These limitations highlight the need for future studies with broader clinical variables, longitudinal follow‑up, functional assays, and larger, more diverse cohorts to better characterize the interplay between virulence genotypes and periodontal disease severity.

Furthermore, targeted therapeutic approaches, including the use of gingipain inhibitors or agents that inhibit fimbrial adhesion, offer promising prospects for the development of personalized periodontal treatment.

## 5. Conclusion

In conclusion, this study demonstrated that *fimA* type II and type IV were the most prevalent fimbrial genotypes, and *kgp-I* and *rgpA* type A were the dominant gingipain genotypes in periodontitis cases. Significant correlations were observed between *fimA* types II and IV and gingipain genotypes *kgp-I*, *kgp-II*, and *rgpA* type A, and their combined presence was strongly associated with greater probing depth and clinical attachment loss, supporting the hypothesis that these virulence factors act synergistically to influence disease severity. Furthermore, *fimA* type II showed the highest prevalence across all bacterial load percentiles, reinforcing its role in pathogenic potential. These findings address the primary aim by confirming genotype associations with *P. gingivalis* levels and periodontitis, and the secondary aim by demonstrating their relationship with clinical parameters. Integrating microbial virulence profiling with host immune characterization may enhance risk stratification and guide personalized periodontal care strategies.

## Author Contributions

Manohar Kugaji contributed to the data collection, conducting experimental procedures, data interpretation, data analysis, and writing original draft. Kishore Bhat and Uday Muddapur contributed to the conceptualization, review, and editing. Ram Surath Kumar played a role in statistical analysis. Suman Kumar Ray contributed to the table and figure preparation. Eswar Kandaswamy and Vinayak Joshi contributed to the conceptualization, original draft preparation, review, and editing.

## Funding

The authors declare that they have not received funding.

## Disclosure

All authors have read and approved the final manuscript.

## Ethics Statement

The study was approved by the Institutional Review Board of Maratha Mandal’s NGH Institute of Dental Sciences and Research Centre, Belagavi (Certificate no. 2016/819). Informed consent was obtained from all subjects involved in the study.

## Conflicts of Interest

The authors declare no conflict of interest.

## Data Availability

The data used/analyzed in this study is available from the corresponding author on request.
